# Recent Advances in Quantum Dot-Based Lateral Flow Immunoassays for the Rapid, Point-of-Care Diagnosis of COVID-19

**DOI:** 10.3390/bios13080786

**Published:** 2023-08-03

**Authors:** Seyyed Mojtaba Mousavi, Masoomeh Yari Kalashgrani, Ahmad Gholami, Navid Omidifar, Mojtaba Binazadeh, Wei-Hung Chiang

**Affiliations:** 1 Department of Chemical Engineering, National Taiwan University of Science and Technology, Taipei City 106335, Taiwan; d10906820@mail.ntust.edu.tw; 2 Biotechnology Research Center, Shiraz University of Medical Science, Shiraz 71468-64685, Iran; masoomeh.yari.72@gmail.com (M.Y.K.); gholami@sums.ac.ir (A.G.); 3 Department of Pathology, School of Medicine, Shiraz University of Medical Sciences, Shiraz 71468-64685, Iran; omidifarn@sums.ac.ir; 4 Department of Chemical Engineering, School of Chemical and Petroleum Engineering, Shiraz University, Shiraz 71557-13876, Iran; binazadeh@shirazu.ac.ir

**Keywords:** COVID-19, detection, lateral flow immunoassay, quantum dots, point-of-care testing, performance

## Abstract

The COVID-19 pandemic has spurred demand for efficient and rapid diagnostic tools that can be deployed at point of care to quickly identify infected individuals. Existing detection methods are time consuming and they lack sensitivity. Point-of-care testing (POCT) has emerged as a promising alternative due to its user-friendliness, rapidity, and high specificity and sensitivity. Such tests can be conveniently conducted at the patient’s bedside. Immunodiagnostic methods that offer the rapid identification of positive cases are urgently required. Quantum dots (QDs), known for their multimodal properties, have shown potential in terms of combating or inhibiting the COVID-19 virus. When coupled with specific antibodies, QDs enable the highly sensitive detection of viral antigens in patient samples. Conventional lateral flow immunoassays (LFAs) have been widely used for diagnostic testing due to their simplicity, low cost, and portability. However, they often lack the sensitivity required to accurately detect low viral loads. Quantum dot (QD)-based lateral flow immunoassays have emerged as a promising alternative, offering significant advancements in sensitivity and specificity. Moreover, the lateral flow immunoassay (LFIA) method, which fulfils POCT standards, has gained popularity in diagnosing COVID-19. This review focuses on recent advancements in QD-based LFIA for rapid POCT COVID-19 diagnosis. Strategies to enhance sensitivity using QDs are explored, and the underlying principles of LFIA are elucidated. The benefits of using the QD-based LFIA as a POCT method are highlighted, and its published performance in COVID-19 diagnostics is examined. Overall, the integration of quantum dots with LFIA holds immense promise in terms of revolutionizing COVID-19 detection, treatment, and prevention, offering a convenient and effective approach to combat the pandemic.

## 1. Introduction

**Figure 1:**
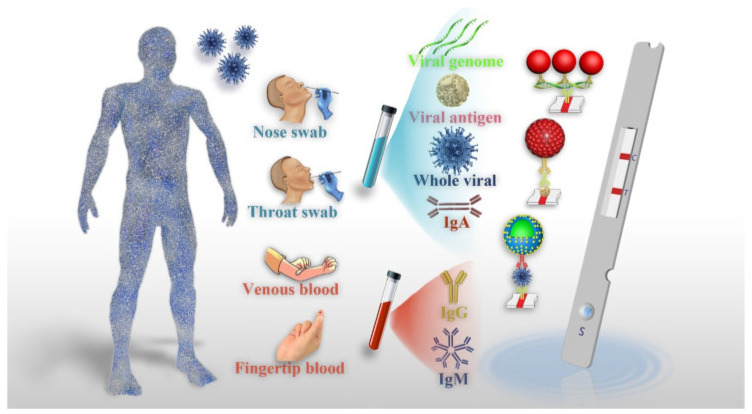
Schematic representation of a POC detection method for COVID-19 using LFIA. Reproduced with permission from Ref. [9]. © 2021 Elsevier B.V. All rights reserved (License Number: 5566111027872).

The emergence of the coronavirus disease 2019 (COVID-19) in Wuhan, China, towards the end of 2019, swiftly evolved into a global pandemic, resulting in significant consequences for human health and wellbeing [1,2,3]. The rapid identification and analysis of the responsible microorganism’s genetic makeup are crucial for developing diagnostic tests, drugs, and vaccines to control and minimize the impact of emerging infectious diseases. This is particularly significant for viral diseases like COVID-19, which exhibit high transmissibility, pathogenicity, and virulence. The real-time reverse transcriptase polymerase chain reaction (rRT-PCR) assay, a method that amplifies and detects specific viral genetic sequences within a few hours, has proven to be a vital and indispensable diagnostic tool in recent times. However, it should be noted that the rRT-PCR assay still requires significant labor and expertise, and it is only accessible in hospitals equipped with a qualified microbiology laboratory [4,5,6]. The surge in COVID-19 patients resulting from the widespread community outbreak has placed limitations on the abilities of the rRT-PCR assay. The sensitivity and accuracy of this diagnostic method for COVID-19 are significantly influenced by factors such as specimen collection site, technique, and timing of the disease course. Consequently, there is a need for a simple, user-friendly, and precise diagnostic tools to complement COVID-19 diagnosis, in order to improve patient outcomes, optimize resource allocation, and enhance infection control measures. In the realm of COVID-19 diagnosis, a groundbreaking innovation has emerged in the form of Point-of-Care (POC) lateral flow immunoassays (LFIAs), which harness the power of quantum dots (QDs). This innovative approach offers the simultaneous or separate detection of anti-SARS-CoV-2 antibodies, revolutionizing the diagnostic landscape for COVID-19 (Figure 1) [7,8,9,10]. Point-of-care testing (POCT) plays a crucial role in enabling healthcare professionals to make timely clinical decisions and implement appropriate treatments. POCT has emerged as a crucial diagnostic tool for disease control, especially in areas with limited resources [11,12]. Its affordability has made it widely accessible, and it has been extensively employed in mass screening surveillance programs to effectively contain the transmission of COVID-19. The advantages of POCT include its low cost, rapidity, simplicity, efficiency, and effectiveness in identifying specific disease biomarkers. In recent years, several innovative POCT diagnostic strategies have emerged [13]. A notable approach in diagnostic strategies is the utilization of the lateral flow immunoassay (LFIA). LFIA combines labeled immunoassays with chromatography, leveraging capillary forces to facilitate the movement of the analyte. The membrane surface is immobilized with specific recognition elements, representing binding moieties that can detect various analytes, including allergens [14,15]. LFIAs exhibit several prominent features [16]. First, they exhibit a rapid reaction speed, delivering results within a matter of minutes. This quick turnaround time is crucial for facilitating prompt decision-making and interventions. Second, LFIAs offer the advantage of automatically separating target analytes from biological samples, eliminating the need for complex additional steps. This simplifies the testing process and reduces the risk of human error. Lastly, LFIAs are able to adapt to diverse outdoor environments, making them suitable for deployment in various settings. One of the key advantages of these diagnostic tests is that they do not necessitate highly skilled personnel to operate specialized equipment or carry out complex analytical procedures. This further enhances their accessibility and practicality. Although commercial LFIAs were initially designed for the detection of human chorionic gonadotropin, their applications have considerably expanded since then [17]. The LFIA methodology has garnered substantial attention across multiple disciplines, owing to its versatility and potential for rapid on-site testing. This is primarily because LFIAs effectively fulfill the ASSURED criteria (Deliverable to end-users, Rapid/Robust, Equipment-free, User-friendly, Sensitive, Specific, and Affordable) for point-of-care testing [18]. Subsequently, a wide range of LFIAs have been developed for disease diagnosis, drugs, pathogens, food analysis, and biomarkers, as well as for toxins and the identification of chemical contaminants [19,20,21]. Gold nanoparticles are typically used as labels in traditional LFIAs, which rely on the nanoparticles’ localized surface plasmon resonance effect. This method, however, only provides qualitative, visually-assessed results, making the labeling system subjective and error-prone. Additionally, its usefulness for analyzing analytes at high concentrations is restricted [22]. However, the widespread application of organic fluorescent dyes is hampered by issues like photobleaching, low quantum yield, and poor stability. These challenges have prompted the exploration of alternative labels to replace gold nanoparticles, in order to advance and enhance the application of LFIAs. Examples of such labels include fluorescent reporters [23,24,25], color latex [26], and magnetic nanoparticles [27]. Among these alternatives, QDs [23,28,29] have emerged as the most promising fluorescent reporters [30,31]. This is primarily due to their inherent properties, including prolonged fluorescence lifetimes, excellent stability, high extinction coefficients, and high quantum yields. The combined characteristics of QDs make them an exceptional choice as reporters for the advancement of highly sensitive LFIAs that are capable of simultaneously quantifying multiple analytes. Recent studies have highlighted the utilization of QD-based LFIAs, which utilize antigen–antibody reactions, to detect and measure the concentrations of diverse analytes such as tumor markers [32], toxins [33], and viruses [34]. This technology offers numerous advantages, including swift detection, excellent stability, and cost-effectiveness, coupled with a user-friendly methodology. For the detection of ochratoxin A in maize, Duan’s team has published an important step forward, detailing the development of a size-dependent competitive immunochromatographic assay using QD nanobeads. This assay’s efficacy was proved by its impressive sensitivity and the quantitative data it supplied [35]. In another investigation, Chen et al. [36], used novel quantum dot-doped polystyrene nanoparticles to create a lateral flow test strip system. This system allowed for the detection of a carcinoembryonic antigen and a cytokeratin-19 fragment in human serum. A QD-based LFIA for the detection of puerarin in both biological and water samples was recently introduced in a publication by Qu and colleagues [33,37]. One of the key advantages of QD-based LFAs is their enhanced sensitivity compared with traditional LFAs. Quantum dots emit intense fluorescent signals upon excitation, allowing for the detection of low concentrations of viral antigens. Consequently, this improved sensitivity leads to the early detection of COVID-19 infections, even during the asymptomatic phase, reducing the risk of transmission and enabling timely intervention. Moreover, QD-based LFAs demonstrate excellent stability and photostability, ensuring the accuracy and reliability of test results over an extended period. Furthermore, the multiplexing capability of QD-based LFAs enables the simultaneous detection of multiple viral antigens, including SARS-CoV-2 variants. This feature contributes to a comprehensive diagnosis, guiding appropriate clinical decisions and providing critical epidemiological data for tracking viral mutations. This study showcased the versatility of QD-based LFIAs when detecting specific compounds, even in complex matrices. Although there have been numerous reports highlighting the potential of QD-based LFIAs in various applications, including disease diagnostics, it is worth noting that these assays have not been extensively employed for identifying allergy disorders. The purpose of this article is to review recent advances in quantum dot-based lateral flow immunoassays for the rapid, point-of-care diagnosis of COVID-19. Additionally, COVID-19 and related biomarkers, point-of-care testing, lateral flow immunoassays, and quantum dots were evaluated. Finally, point-of-care LFIA for the detection of COVID-19 QD-based LFIA point-of-care, and the performance of the QD-based LFIA as point-of-care testing for COVID-19 detection, were studied.

## 2. COVID-19 and S/N Proteins

**Figure 2:**
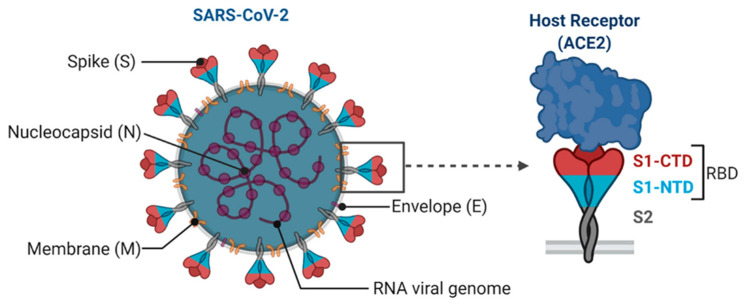
Basic principles of the novel SARS-CoV-2 coronavirus. Reproduced with permission from Ref. [54].

Coronaviruses are a diverse family of viruses, classified into four genera (α, β, γ, δ), based on genotyping and serology. α and β coronaviruses primarily infect mammals and they can cause diseases in both humans and animals, whereas γ and δ coronaviruses are more commonly found in birds. The novel coronavirus SARS-CoV-2, responsible for the ongoing pandemic, belongs to the β-coronavirus genus [38]. Among the seven coronavirus species known to be transmitted through human contact, NL63, OC43, 229E, and HKU1 generally cause milder illnesses. However, the three β-coronaviruses, including SARS-CoV, MERS-CoV, and SARS-CoV-2, exhibit high pathogenicity, which can lead to severe and often fatal outcomes [39,40,41]. The contagiousness of a viral infection is commonly assessed using a crucial parameter known as the reproduction number (R0), which quantifies the rate of virus transmission [42]. SARS-CoV-2 has a single-stranded positive-sense RNA genome approximately 30,000 nucleotides long. It is a relatively large virus with a diameter ranging from 70–140 nm, and it exhibits prominent spikes on its surface, measuring about 9–12 nm in length. These spikes play a crucial role in attaching to, and entering, host cells [40,43,44]. The genome of SARS-CoV-2 encodes four structural proteins, as follows: spike protein (S), nucleocapsid protein (N), coat protein (E), and membrane protein (M) (Figure 2) [45]. The five primary open reading frames (ORFs) in the genome are organized in a manner that is consistent with the 5′ untranslated region (5′ UTR)-replicase complex [46]. Researchers such as Tang et al. have identified 149 mutation sites in the SARS-CoV-2 genome. These mutations have given rise to two distinct viral types, the L-type and S-type, which comprise 70% and 30% of the viral population, respectively. These variations have contributed to the heightened contagiousness of the virus [47].

The S/N protein, also known as the spike protein, plays a crucial role in detecting COVID-19. This protein is present on the surface of the SARS-CoV-2 virus, which causes the COVID-19 disease. The S/N protein has distinctive characteristics that make it an ideal target for detection methods. It is a glycoprotein that protrudes from the surface of the SARS-CoV-2 virus, and it is responsible for binding and entering human cells. The S/N protein consists of two subunits, S1 and S2. The S1 subunit contains the receptor-binding domain (RBD) that specifically interacts with the ACE2 receptor on human cells, facilitating viral entry. This interaction is the primary target when neutralizing antibodies and conducting therapeutic interventions. The S2 subunit aids in membrane fusion, allowing the virus to enter the host cell and initiate infection. It contains a fusion peptide and two heptad repeat regions (HR1 and HR2), which undergo conformational changes during fusion. Due to its vital role in viral entry and infection, the S/N protein is a primary target for diagnostic tests, such as antigen tests and serological assays. These tests detect the presence of S/N protein or its antibodies in patient samples, enabling the identification and diagnosis of COVID-19 cases [48,49,50,51,52,53].

### Current Diagnostic Approaches for the Detection of COVID-19

**Figure 3:**
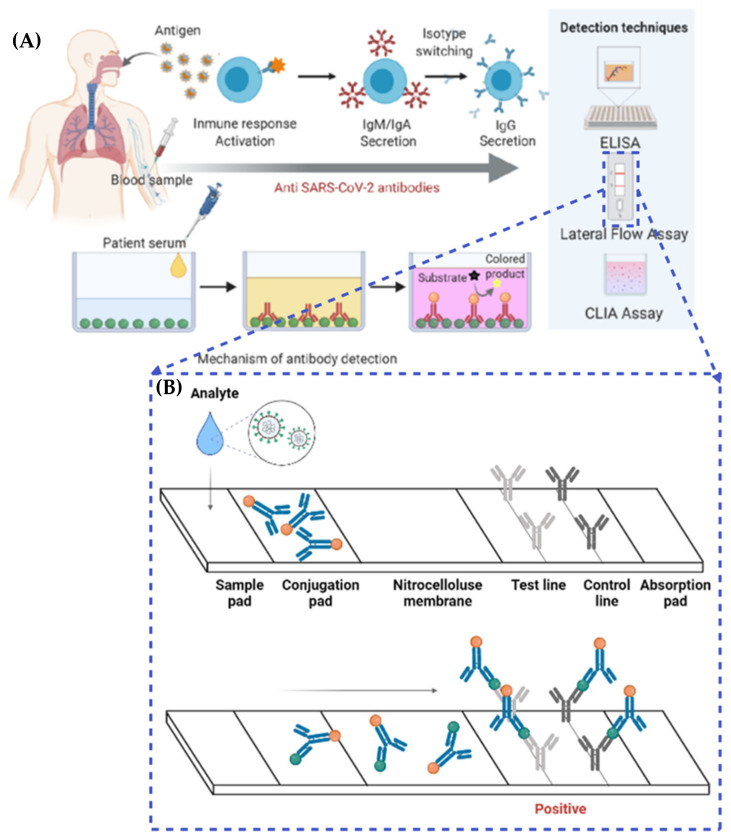
(**A**) Indirect detection methods to detect anti-SARS-CoV-2 antibodies. Reproduced with permission from Ref. [61]. (**B**) Lateral flow assay (LFA) detects SARS-CoV-2 antigens. Reproduced with permission from Ref [62]. © 2022 The Author(s). Published by Elsevier B.V. on behalf of King Saud University.

A variety of diagnostic methods have been developed to detect viral infections, taking into account the nature of the virus, its characteristics, and the collected patient samples. In such cases, there is a need for sensitive, equipment-free, user-friendly, rapid, specific, accessible platforms that are cost-effective and robust. Point-of-care testing (POCT) fits these criteria, as it offers a convenient and self-contained approach for detecting SARS-CoV-2 viral infections. Furthermore, POCT does not rely on highly trained personnel or extensive healthcare facilities, making it suitable for on-site triage. It also eliminates the need for complex sample preparation, and it reduces the overall workload required to perform the test [55]. Mujawar et al [56]. investigated the use of point-of-care diagnostics for COVID-19 using miniaturized nanoenabled sensors in conjunction with AI and the Internet of Things (IoT). Their study highlighted the promising benefits of AI in terms of combating global pandemics, emphasizing its potential impact on disease detection and control [56]. In light of the rapidly escalating global pandemic, there is an urgent requirement for point-of-care testing (POCT) platforms to effectively combat the spread of the virus. These POCT devices serve a critical role in monitoring asymptomatic individuals and detecting SARS-CoV-2. Mass testing on a significant scale is essential to prevent widespread transmission throughout the population. Recently, POCT platforms such as lateral flow immunoassays (LFIA) have emerged as simpler, rapid, and cost-effective solutions for diagnosing SARS-CoV-2. However, these platforms face challenges that are related to inadequate sensitivity, selectivity, and overall reliability [57,58,59,60]. Moreover, indirect methods of detection aim to identify antibodies against SARS-CoV-2, which are generated by the host’s immune cells following infection. This approach involves utilizing recombinant viral proteins as bait to capture the host’s antibodies. It is important to note that due to the time required for antibody production, a positive outcome indicates a previous infection, but does not necessarily indicate a current infection. Methods for detecting antibodies include chemiluminescent immunoassays (CLIA), lateral flow assays (LFA), and enzyme-linked immunosorbent assays (ELISA) (Figure 3A) [61]. By moving samples from the sample pad to the conjugation pad, the LFA technique can identify SARS-CoV-2 antigens in infected people. Specific and non-specific conjugated antibodies both bind to the antigens. Positive samples are indicated when the resultant complexes pass through the nitrocellulose membrane and bind to anti-SARS-CoV-2 antibodies, resulting in the development of color. Thus, an analyte flow control line that generates color can be used to verify the proper flow of the analyte (as shown in Figure 3B) [62].

## 3. Point-of-Care Testing (POCT)

To facilitate the expansion of testing efforts, the advancement of quick and easily accessible molecular diagnostic tests at point of care (POC) is crucial. These tests should possess a level of sensitivity and specificity that is comparable to current gold standard techniques. If they achieve this, they can play a significant role in enhancing testing capabilities. POC devices have the potential to provide convenient access to information regarding the presence of the virus, as well as the host’s response, such as the presence of antibodies. These devices can be utilized in various nonlaboratory settings, and they offer rapid results, thereby expediting the process. Deploying testing solutions beyond centralized laboratories, particularly at primary or urgent care facilities, is a vital step towards swiftly detecting and identifying cases of COVID-19. This approach can contribute to preventing the further transmission of the virus within the community. The utilization of point-of-care devices presents several advantages. Firstly, they allow for the use of portable and cost-effective instrumentation. This enables easier access to testing resources while keeping expenses under control. Secondly, these devices eliminate the need to transport samples to a clinical laboratory for analysis. This not only saves time, but it also reduces logistical complexities. Thirdly, point-of-care devices streamline sample processing procedures, making the overall testing process more efficient. Fourthly, they make use of samples like saliva or anterior nasal swabs, which can be collected without the need for trained personnel. This simplifies the collection process and improves accessibility to testing for individuals. Lastly, these devices have the ability to measure various entities such as viruses, antigens, and antibodies in both symptomatic and asymptomatic patients. This comprehensive approach allows for the more accurate determination of individuals who would benefit from clinical care or require quarantine. The selection of appropriate diagnostic solutions depends on factors such as the desired throughput, portability, cost, and the regulatory approval process [63,64,65].

## 4. Lateral Flow Immunoassay (LFIA)

**Figure 4:**
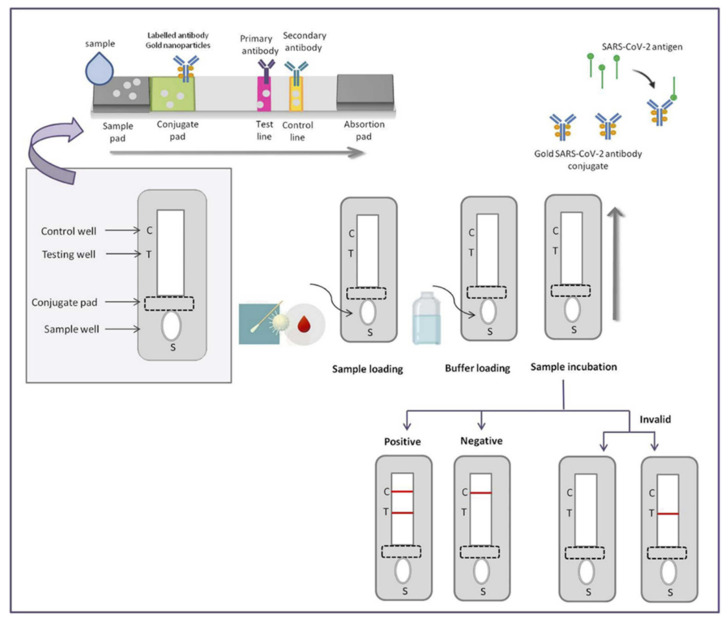
Schematic of the lateral flow immunoassay (LFIA) for the detection of COVID-19. Reproduced with permission from Ref. [71]. © 2022 The Author(s). Published by Elsevier Masson SAS.

In contrast to many immunological assays, the LFIA stands out as a rapid point-of-care test that delivers quick results without the need for prior training or laboratory facilities. These unique characteristics have made LFIA devices highly appealing for a wide range of applications beyond the detection of infectious agents. Their versatility extends to fields such as environmental sciences, drug research, food analysis, and various clinical investigations. The LFIA operates through a straightforward immunoassay approach, primarily relying on the accumulation of antibodies or antigens conjugated to reporter molecules. These molecules are strategically positioned on specific areas of the test strip, known as the test and control regions. By depositing capture molecules, the LFIA enables the detection of analyte–conjugate complexes on the membrane of the strip. This simplified process allows for the efficient and accurate identification of the target analyte, facilitating the overall testing procedure [66,67,68,69,70]. A schematic of the lateral flow immunoassay (LFIA) for the detection of COVID-19 is shown in Figure 4. Table 1 shows the applications of LFIA in the clinical field.

### 4.1. Principles of LFIA

**Figure 5:**
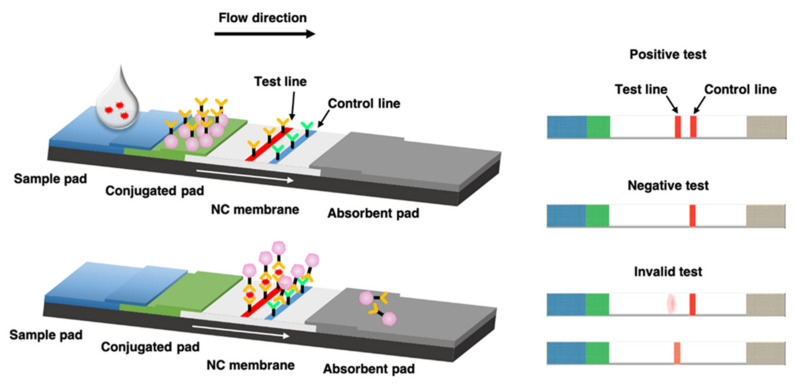
Schematic illustration of LFIA test based on antigen detection. Reproduced with permission from Ref. [91].

The fundamental principle of the LFIA is simple. Through capillary action, a liquid sample or an extract containing the specific analyte of interest is guided across multiple zones of polymeric strips. This process occurs naturally, without the need for external forces or interventions. As a result, the LFIA enables the detection and analysis of the target analyte in a convenient and user-friendly manner. These strips are coated with molecules capable of interacting with the analyte. Figure 5 depicts the process as follows. The initial step involves the application of the sample onto the adsorbent sample pad located at one end of the strip. This pad is enriched with surfactants and buffer salts, which effectively prepares the sample for its interaction with the detection system. Utilizing the sample pad, the analyte within the sample is aided in its attachment to the capture reagents present on both the membrane and the conjugates. This ensures that the analyte exists in the necessary conditions to effectively interact with the detection components of the LFIA system. The processed sample progresses through the conjugate release pad, which consists of antibodies specifically designed to target the analyte of interest. Fluorescent or colored particles, such as latex microspheres and colloidal gold, are commonly used to conjugate the antibodies. These conjugates play a vital role in the process. As the sample advances along the strip, the antibodies, along with the conjugated particles which have bound to the target analyte, travel in parallel. They eventually reach the detection zone, which encompasses a porous membrane predominantly composed of nitrocellulose. Within the detection zone, specific biological components are immobilized in separate lines, enabling the detection and analysis of the target analyte. These components mainly consist of antibodies or antigens that are specific to the analyte being tested. The main purpose of these components is to interact with the analyte that is attached to the conjugated antibody. When the sample analyte is recognized, it initiates a unique reaction at the test line. Conversely, a response on the control line indicates that the liquid is flowing correctly through the strip. The visual interpretation of the test results involves observing the lines, which appear in varying intensities. Alternatively, a specialized reader can be used for more precise measurements. To enable the simultaneous testing of multiple analytes under the same conditions, an array format can be utilized. This involves incorporating additional test lines on the strip so that antibodies specific to different analytes are immobilized [82,83]. In contrast, for semi-quantitative assays, multiple test lines can be employed, each loaded with the same antibody. The assay described, known as the “ladder bars” assay, functions in accordance with the principle of sequentially capturing colorimetric conjugate–antigen complexes. Each immobilized antibody on the successive lines captures these complexes, and the number of lines appearing on the strip directly correlates with the concentration of the measured analyte [84,85,86,87]. This step-by-step process allows for quantitative analysis, and it provides a visual representation of the analyte concentration. The movement of liquid through the device is facilitated by the capillary force exerted by the strip material. To sustain this flow, an absorbent pad is affixed at the end of the strip. The absorbent pad serves the purpose of absorbing excess reagents and preventing any backward flow of the liquid. LFIAs can be categorized into two main formats: direct and competitive. In the field of diagnostics, the direct format is utilized for detecting larger analytes. For example, it is employed in the detection of the p24 antigen in the human immunodeficiency virus (HIV) test [88,89]. Additionally, the direct format is valuable for analytes that possess multiple antigenic sites, such as human chorionic gonadotropin (hCG) used in pregnancy tests [90]. These applications highlight the effectiveness of the direct LFIA format in specific diagnostic scenarios. An illustration of such an assay is the hCG test, which follows a sandwich-based approach. In this method, the target analyte is securely immobilized between two perfectly matched antibodies, forming a robust diagnostic framework. In the direct testing approach, a positive result is indicated by the presence of the test line, whereas the control line typically contains anti-immunoglobulin antibodies specific to the particular conjugate’s antibody, and that are tailored to the species being tested. Competitive tests, on the other hand, are employed for small molecules with single antigenic determinants that cannot simultaneously bind to two antibodies. In this type of test, the analyte obstructs the binding sites on the antibodies located at the test line, preventing their interaction with the colored conjugate. Consequently, a positive result is denoted by the absence of a signal in the test line and the control line should remain visible regardless of the test outcome.

### 4.2. Components of the LFIA

**Figure 6:**
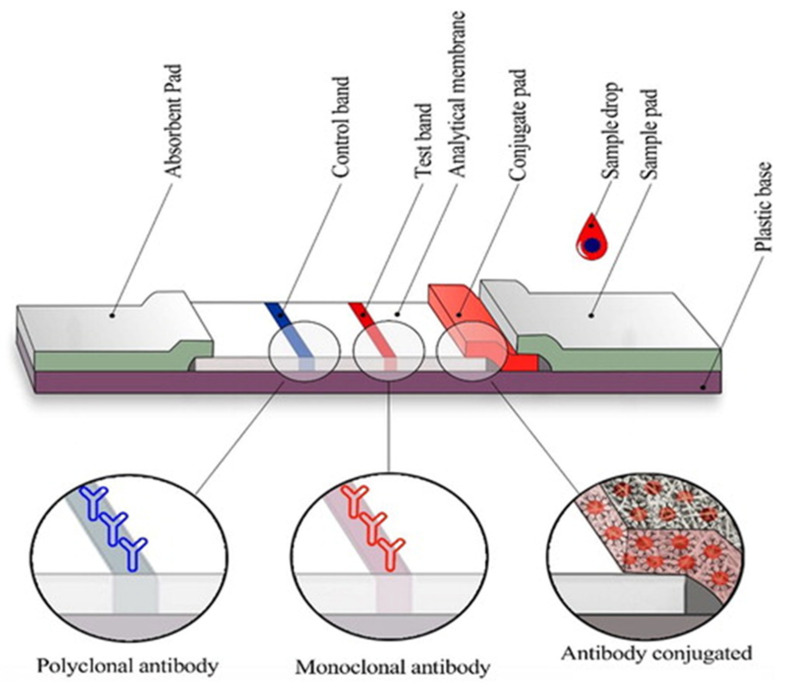
Typical configuration of a lateral flow immunoassay test strip. Reproduced with permission from Ref. [107]. Copyright © 2023 Informa UK Limited.

The manufacturing of LFIA devices often encounters significant challenges arising from the intricate nature of the device itself. With numerous elements involved, issues can arise due to material incompatibility, faulty connections between overlapping components, or less-than-ideal material characteristics. When developing LFIAs, the primary emphasis has largely been placed on identifying the most appropriate detection method, or selecting the optimal antibody or antigen. However, it is crucial to give due consideration to every component of the test, encompassing fundamental elements like the adhesive strip, cover tape, and backing card, to ensure the creation of a reliable and superior-quality product [92,93,94]. The overlapping membranes that are firmly linked to a backing card comprise the conventional LFIA test strip, as seen in Figure 6. This design provides several advantages, including improved ease of handling and enhanced stability throughout the testing process. LFIA devices consist of several components that work together to enable accurate and efficient testing [95,96,97]. The sample pad is responsible for distributing and guiding the sample toward the conjugate pad. It is typically impregnated with surfactants, buffer salts, and proteins to regulate the flow rate and optimize compatibility with the detection system. The sample pad also acts as a filter, removing unwanted substances such as red blood cells and particulate matter. The conjugate pad retains the detector particles and maintains their functionality until the test is conducted. It is enriched with a conjugate buffer that contains carbohydrates, such as sucrose. These carbohydrates serve as preservatives, ensuring the longevity and viability of the detector particles. They also act as resolubilization agents, aiding in the reconstitution of dried detector particles during the assay process [98,99,100,101]. The membrane is a critical element in LFIA devices, and it is typically composed of nitrocellulose. It plays a paramount role in binding and immobilizing proteins effectively for subsequent selection, reaction, and detection processes. The membrane may have different pore sizes, and its capillary flow time is evaluated to determine its effectiveness. The absorbent pad draws the fluid through the membrane and collects the processed liquid. It often contains cellulose filters, which enable the use of larger sample volumes and promote enhanced fluid flow. The integration of cellulose filters within the absorbent pad enhances the overall sensitivity of the test. The label is a crucial component that enables the detection of the target analyte. Colloidal gold and latex are commonly used labels in LFIA devices. Colloidal gold offers stability, a wide dynamic range, efficient conjugation, and minimal non-specific binding. Latex labels provide versatility and stability, allowing for various detection methods and applications. These components, along with the careful design and purification of antibodies, contribute to the overall performance, reliability, and accuracy of LFIA devices. Each component plays a specific role in sample distribution, detection, and analysis, ensuring optimal test results [102,103,104,105,106].

### 4.3. Advantages and Disadvantages of LFIAs

**Figure 7:**
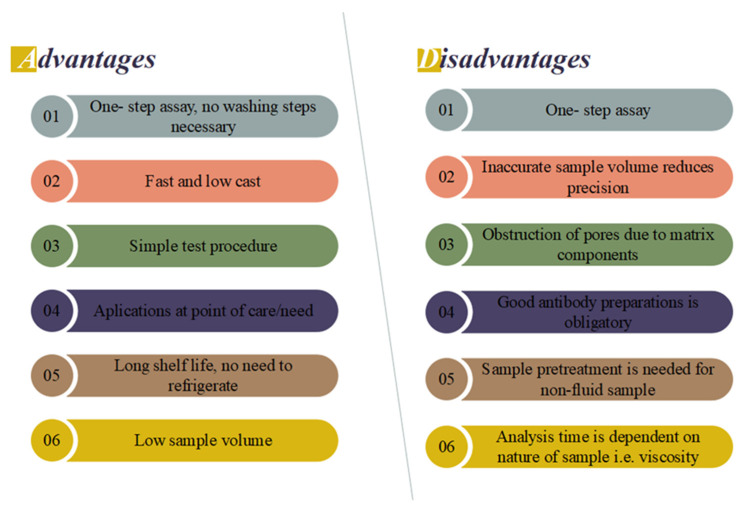
Advantages and disadvantages of the LFIA.

Numerous LFIAs are specifically designed for point-of-care/need applications, offering cost-effective, speedy, and user-friendly testing solutions that are highly sought after in various industries. Despite this, regulatory bodies will frequently insist on the results of an experiment being confirmed via an independent approach. As a consequence of this, LFIA tests are largely appropriate for use as initial screening procedures at POC, or when necessary. Due to their longer shelf life, and the fact that they do not need to be refrigerated, they are particularly well-suited for use in countries that are still economically developing. The visual outcomes of LFAs are typically straightforward and discernible, eliminating the necessity for specialized equipment. Figure 7 provides a comprehensive overview of the pros and cons of LFAs. Ongoing research endeavors are dedicated to tackling the prominent limitations of LFAs, particularly in terms of obtaining quantitative results. Scanners or cameras equipped with dedicated software can digitize the data, facilitating result documentation. However, advancements in technology may impact the expenses associated with the apparatus and the analysis duration [78,108,109].

## 5. POC LFIA for the Detection of COVID-19

The LFIA stands out as an appealing detection tool that possesses all the necessary qualities for effective colorimetric assays. A widely employed lateral flow device comprises four essential elements: the sample pad, conjugate pad, detection membrane, and absorbent pad. These components are thoughtfully designed to enable the seamless flow of samples through channels, leveraging the capillary action mechanism [59,68]. The sample pad consists of glass and/or cellulose materials, serving as the initial point of application for the detection sample. An essential component of the LFIA setup is the sample pad, which plays a pivotal role in ensuring the seamless and uninterrupted transportation of samples to other crucial assay components. Positioned alongside is the conjugate pad, a reservoir that houses labeled biorecognition molecules. When the running sample interacts with the conjugate pad, it triggers the release of the conjugate labels. In a traditional LFIA configuration, these labels often take the form of colloidal nanoparticles, such as AuNPs (gold nanoparticles) or AgNPs (silver nanoparticles). This synchronized interaction sets the stage for the subsequent stages of the assay, enabling the detection and analysis of the target analytes with precision and accuracy. The sensitivity of the LFIA is significantly influenced by the presence of labeled conjugates. The detection membrane holds significant importance in determining the overall sensitivity of the LFIA. The membrane utilized in the LFIA is strategically designed to incorporate test lines and control lines, forming a robust framework for efficient binding with capture probes, including antigens, antibodies, and other pertinent components. These well-defined lines provide the necessary foundation for accurate and reliable detection. In the final stages of the assay, the adsorbent pad, acting as a sink-like structure, is connected to the detection membrane. This integration serves multiple purposes, such as maintaining the optimal flow rate of the sample and preventing any undesired backward flow. Moreover, the adsorption capacity of the sample holder plays a crucial role in influencing the assay results, underscoring the significance of this component in achieving precise and dependable outcomes [110]. The traditional LFIA method offers qualitative or semi-quantitative outcomes through a straightforward process that relies on color visualization, eliminating the need for additional equipment. A positive result is indicated when color is detected on both the test line and control line, providing a clear indication of the presence of the target analyte. Conversely, a negative result is established when only color is observed on the control line, suggesting the absence of the target analyte in the sample [111]. One of the key advantages of the LFIA is its accessibility and convenience, as it does not necessitate well-equipped laboratories or extensively trained personnel to perform the tests. This user-friendly aspect ensures that the LFIA can be deployed in various settings, enabling swift and efficient testing without unnecessary logistical constraints. It provides rapid and reliable results, ensuring efficiency in diagnostic procedures. Furthermore, the versatility of LFIA tests when detecting pathogens extends to a diverse array of clinical samples, encompassing serum, plasma, whole blood, saliva, and more. This versatility positions LFIA as an optimal diagnostic assay for point-of-care testing, particularly in emergency departments and resource-limited environments. In the realm of SARS-CoV-2 antibody testing, the LFIA has emerged as a rapid and precise method for point-of-care diagnostics. It empowers healthcare professionals to swiftly identify specific antibodies like IgM, IgG, and IgA in whole blood, serum, and plasma, providing distinct and discernible results for targeted antibody detection [112]. A shining example of the LFIA’s potential lies in the groundbreaking work of Li et al., who successfully engineered a LFIA using the remarkable properties of gold nanoparticles. This ingenious creation facilitated the simultaneous detection of IgM and IgG antibodies within clinical samples, delivering results within a matter of minutes [8]. Undoubtedly, the LFIA unveils a vast realm of applications, stretching far beyond the boundaries of traditional clinical settings, offering a versatile and invaluable tool for both clinical and non-clinical domains alike. However, it is not without limitations, as it may have reduced sensitivity when dealing with higher analyte concentrations and it may exhibit poor reproducibility, which are notable drawbacks that need to be addressed. Furthermore, the LFIA offers the advantage of delivering rapid results within a short timeframe, typically ranging from 10 to 30 min. It is also characterized by its user-friendly nature, making it accessible to a wider range of individuals. However, it is important to note that the LFIA primarily provides qualitative information and may not always offer sufficient accuracy. As a result, there is an urgent need for quick and accurate diagnostic techniques to improve SARS-CoV-2 viral testing capabilities. These methods should be capable of high-throughput diagnosis while eliminating the requirement for extensive technical expertise or advanced tools [37,113].

**Figure 8:**
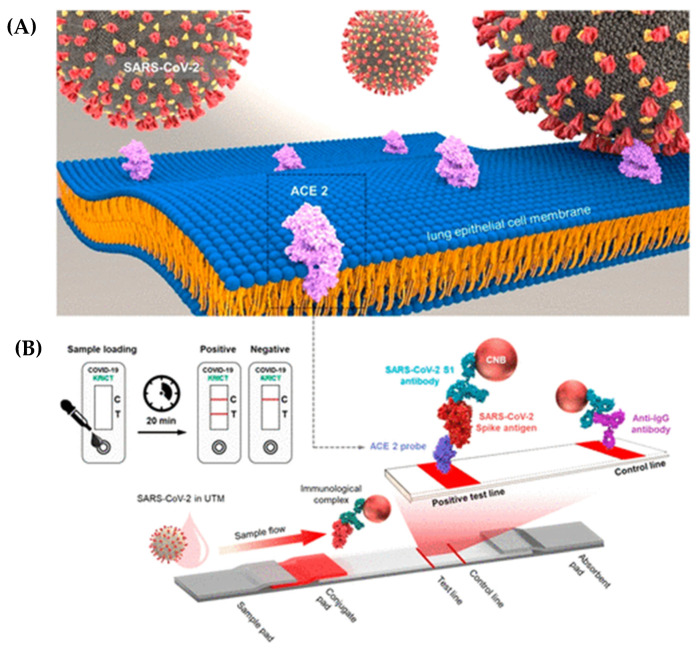
An LFIA (Lateral Flow Immunoassay) system based on the cellular receptor, ACE2. (**A**) The schematic showcases the recognition process between ACE2 and SARS-CoV-2. ACE2, which is a type 1 membrane protein expressed in the lung, heart, kidneys, and intestine, serves as the cellular receptor for the virus. (**B**) Showcase of the components of the ACE2-based LFIA. The LFIA system consists of a sample pad, conjugate pad, nitrocellulose membrane, and absorbent pad. The nitrocellulose membrane features a test line that contains ACE2, enabling the detection of the SARS-CoV-2 spike antigen. In addition, the control line utilizes an anti-IgG antibody for validation purposes within the LFIA system. Reproduced with permission from Ref. [118]. Copyright 2020 Elsevier (License Number: 5566101395195).

Zeng et al. [114] developed a single LFIA test kit for blood samples that could detect both IgM and IgG antibodies at once using an immunosensor chip. The gold nanoparticles tagged with S-protein and rabbit IgG antibodies were immobilized onto the sample pad to create the LFIA kit. Antibodies specific to each test line (M line, G line, and control line) were used in their creation. The presence of IgM or IgG antibodies in a clinical sample is indicated by a red line on either the M line or the G line in combination with the C line. Remarkably, this method achieves antibody detection within the short timeframe of 15 min. In addition, the presence of a red color on both the M line and G line, along with the C line, signifies the simultaneous presence of both antibodies in the sample. To validate its performance, the researchers conducted tests using 80 samples from patients, comprising both positive and negative cases. The results revealed that the kit demonstrated a sensitivity of 85.29% when detecting the target antibodies. Impressively, the specificity of the kit was found to be 100%, indicating its high accuracy in correctly identifying the absence of the antibodies. Notably, the researchers found that when identifying either IgG or IgM alone, the immunoassay’s sensitivity was substantially reduced. However, there was a significant increase in sensitivity when both antibodies were present in the sample, underscoring the importance of detecting both simultaneously [114]. Embarking on a quest for scientific enlightenment, Guedez-Lopez and colleagues attempted to validate three lateral flow immunoassays—Sienna, Wondfo, and Prometheus. They sought to unravel the secrets held within serum specimens, regarding the presence of IgM and IgG antibodies and how they work against the notorious SARS-CoV-2 disease. In this thrilling exploration, Sienna, Wondfo, and Prometheus emerged as steadfast contenders, each exhibiting their unique prowess in the realm of detection. Sienna showcased an impressive overall sensitivity of 64.4%, whereas Wondfo, with its distinct attributes, demonstrated a commendable sensitivity of 45.2%. However, it was Prometheus that reigned supreme in this triad, boasting an astonishing overall sensitivity of 75.5%. In the quest for specificity, Wondfo and Prometheus continued to shine, with specificities reaching remarkable heights of 75.0% and 81.8%, respectively. Moreover, another contender, Prometheus, displayed a specificity of 12.5%, revealing the multifaceted nature of these immunoassays and the intricate tapestry of their performance. Furthermore, in the third week since disease onset, the sensitivity of these immunoassays was raised to 100%, 83.3%, and 100%, respectively [115]. In a study conducted by Gutierrez et al. [116], ten different immunoassays were thoroughly validated to detect specific antibodies (IgG, IgA, and IgM) against SARS-CoV-2. These immunoassays encompassed three chemiluminescence assays, three LFIA rapid tests, and four ELISA kits. The objective of this research was to assess the performance and accuracy of these immunoassays in the specific detection of SARS-CoV-2 antibodies [116]. Fabian et al. conducted a comprehensive study to validate two commercially available test kits designed for the detection of IgA antibodies targeting SARS-CoV-2: ENZY-WELL SARS-CoV-2 IgA and anti-SARS-CoV-2 IgA EUROIMMUN. The validation process involved analyzing 65 clinical samples, of which, 39 were positive and 26 were negative, and they had already undergone PCR testing. The researchers also assessed the performance of the test kits when detecting IgG or IgM antibodies. The EUROIMMUN Anti-SARS-CoV-2 ELISA Assay was previously evaluated in other studies, and it exhibited excellent sensitivity when detecting IgA and IgG antibodies in samples collected at least 3 and 4 days after a COVID-19 diagnosis, respectively. It also demonstrated good specificity for IgA, and excellent specificity for IgG. Additionally, the study included the evaluation of the ENZY-WELL SARS-CoV-2 IgA test kit, which is a new commercial whole-virus-based ELISA capable of detecting anti-SARS-CoV-2 IgG, IgM, and IgA antibodies. The study highlighted the importance of serological tests for SARS-CoV-2, particularly those detecting antibodies concerning the N or S protein, as they could complement molecular diagnosis, especially in the later stages of the disease or for retrospective studies. These serological tests, along with various commercially available nucleic acid detection kits, contribute to effective COVID-19 diagnosis and monitoring [117]. In a recent study by Lee et al. [118], a new method called the Lateral Flow Immunoassay (LFIA) was introduced for the rapid detection of the S1 protein of SARS-CoV-2 in clinical samples. This innovative approach allows for detection within the short timespan of 20 min. The LFIA strip contains ACE2 receptors which have the potential to bind to the S1 protein of SARS-CoV-2. The sensitivity of this method is remarkably high, capable of detecting the RBD at concentrations as low as 1.0 ng/reaction, and the S1 protein of SARS-CoV-2 at concentrations below 5.0 ng/reaction. The limit of detection (LOD) for this LFIA was determined to be 1.86 × 10^5^ copies/mL in the sample tested. To assess the specificity of the LFIA, the researchers conducted cross-reactivity tests and found no indications of binding between the S1 protein of MERS-CoV, SARS-CoV, and the LFIA strip. The LFIA strip used in this study is based on the cellular receptor ACE2, as depicted in Figure 8A,B [118,119]. 

As the world grappled with the onset of the first reported case of COVID-19, a wave of innovation surged within the healthcare domain. In its wake, a multitude of publications and patents emerged, all united by a shared mission: to engineer diagnostic kits capable of detecting the elusive presence of SARS-CoV-2. This section focuses on discussing notable advancements in the field of biosensors, particularly Lateral Flow Immunoassays (LFIA), which have emerged as promising tools for the diagnosis of SARS-CoV-2. It is important to note that although some of these LFIA methods are currently undergoing research and development, others have already been commercialized [120]. In a groundbreaking demonstration, Cavalera et al. forged new paths by harnessing the power of innovation. Their ingenious approach involved the utilization of a multitarget lateral flow immunoassay (LFIA), seamlessly integrated with a gold nanoparticle conjugate. This ingenious amalgamation led to a remarkable transformation that enhanced both the response and detection capabilities, enabling them to reach unprecedented heights. All antibodies tested had a remarkable overall sensitivity of 94.6%. Upon application of the sample to the LFIA strip, the response time was approximately 20 min. The researchers utilized two test lines that were built especially for the detection of “total antibodies” [76] to identify the antibodies that were specific to SARS-CoV-2. Nicol et al. conducted a comparative review of three immunoassays (that are now available for purchase) in a study that was published in the Journal of Clinical Virology. Their findings revealed that these assays yield more favorable outcomes when testing samples taken 14 days after the onset of symptoms. Interestingly, they observed no notable disparity in sensitivity between the IgM LFIA and IgA ELISA, despite these two assays targeting distinct proteins. Results showed that all three immunoassays provided similarly high clinical performances with regard to the IgG component. It is worth highlighting that the NG-Test proved to be dependable and precise, making it suitable for routine adoption in clinical laboratories [121]. Flower et al. undertook a comprehensive investigation wherein they thoroughly examined the utility of LFIA when conducting a seroprevalence survey on a global scale. To accomplish this, the researchers employed samples that had previously undergone RT-PCR testing, and they administered LFIA assessments in both clinical and laboratory settings. After conducting a thorough analysis and comparing the outcomes, Flower et al. uncovered that LFIA exhibited a sensitivity rate of 84.4% and a specificity rate of 98.6%, showcasing a moderate level of agreement when compared with RT-PCR. However, it was observed that the sensitivity of all LFIA kits was comparatively lower when compared with PCR or ELISA. However, despite this drawback, they exhibited satisfactory levels of specificity, making them viable options for survey studies. The researchers emphasized the significance of conducting additional assessments and advancing the creation of novel, highly refined tests designed specifically for the targeted population as they concluded their findings [122]. In their groundbreaking research, Wen et al. introduced a LFIA strip designed to facilitate the rapid detection of IgG antibodies against SARS-CoV-2. To accomplish this, they made alterations to a conventional LFIA strip that is typically used for detecting SARS-CoV-2 antibodies. The modifications involved integrating bioconjugates of AuNPs, which were aligned with the standard testing process. It is worth mentioning that the test outcomes could be obtained remarkably quickly, within a timeframe of merely 15 to 20 min. During their investigation, the researchers determined that the sensitivity of their LFIA strips reached approximately 69.1%. They also recognized that optimizing various strip parameters, such as membrane selection, Au labeling, blocking solution, *p*-value, and Ag-Ab coupling, among others, could significantly enhance the performance of the LFIA strip. In their conclusion, Wen et al. underscored the significance of focusing future endeavors on enhancing signal detection methods and establishing more resilient quantification systems. By focusing on these aspects, they aim to further enhance the capabilities of LFIA strips for the detection of SARS-CoV-2 antibodies [74]. In their quest to improve the sensitivity of LFIA strips for the detection of SARS-CoV-2, Peng et al. undertook an innovative approach. To conceptually examine and quantify the test sample, the researchers used a straightforward laser optical analysis technique. To enhance sensitivity, they incorporated a photon-counting approach, allowing for more accurate measurements. In their study, Peng et al. utilized AuNPs, which effectively absorbed incident light and facilitated the scattering of signals, thereby boosting the overall sensitivity of the LFIA strip. As a result of these advancements, they achieved the lowest limit of detection (LOD) among all the available LFIA test kits, marking a significant milestone in their research [44,123].

## 6. Quantum Dots (QDs)

High-fluorescence probes that are conjugated with “Quantum dots (QDs)” play a crucial role in the detection and long-term fluorescence imaging of various cellular processes [124,125,126]. QDs have emerged as highly promising markers for immunochromatography. These quantum dots, with diameters ranging from 1 to 100 nm, can be stimulated with a single near-ultraviolet source to produce luminescence that spans across the whole visible spectrum [127,128,129]. Due to their unique and narrow emission spectra, quantum dots are extremely useful as molecular imaging probes. Quantum dots have garnered significant attention as potent reagents in the fight against viral infections. Moreover, collaborative efforts with regard to exploring potentially biocompatible vectors have the potential to facilitate interdisciplinary research and support clinical approaches when combating viruses. Quantum dots can serve as carriers or drug labels, further enhancing their functionality. Multicolor quantum dots have found applications in diverse fields such as flow cytometry [130], biological imaging [131], fluorescence-based ELISA [132,133], and more.

Research has demonstrated the development of an immunochromatographic test capable of simultaneously detecting multiple species within complex sample substrates. The test strip is structured in a “traffic lights” format, featuring three distinct colored lines. Consequently, a straightforward and user-friendly tool has been created, enabling the identification and analysis of substances based on the colors of the existing lines (qualitative analysis). Additionally, the test strip allows for the determination of analyte concentration through quantitative analysis; it relies on the fluorescence intensity of the test line under UV light. This approach provides a comprehensive solution for both qualitative and quantitative assessments of analytes within the sample. To facilitate the creation of multicolor immunochromatography tests, antibodies were engineered to bind to chloramphenicol, streptomycin, and ofloxacin with high selectivity. Each antibody was conjugated with quantum dots that emitted light at different maximum wavelengths: 525 nm, 585 nm, and 625 nm, respectively. Significantly, the immunochromatography tests demonstrated remarkable sensitivity when detecting the presence of antibiotics, boasting detection limits as low as 0.3 ng/mL, 0.12 ng/mL, and 0.2 ng/mL for each respective antibiotic. Comparatively, the data obtained from the study demonstrated detection limits that were 80 to 200 times lower than those achieved using the same antibodies in ELISA assays. These findings highlight the enhanced sensitivity and improved performance of the multicolor immunochromatography tests when detecting and quantifying the target antibiotics. The utilization of this system for analysis eliminates the need for any additional sample pretreatment, making it a straightforward and user-friendly tool. It enables the rapid detection of antibiotics in milk samples simply by dropping the milk onto the designated testing area. Furthermore, the method assay demonstrated an exceptional performance, exhibiting high levels of analyte detection ranging from 92% to 101%. Additionally, it showcased remarkable accuracy with quantitative errors amounting to less than 8% of the mean, thus, it effectively detected the added milk samples. These findings highlight the efficiency, reliability, and accuracy of the system in milk sample analysis [134]. The use of a lateral flow immunoassay technique for competitive and fluorescent detection was also introduced as a novel approach. This technique utilized quantum dots (QDs) in rat plasma to detect triclopyridine, a metabolite of a tick-poisoning organophosphorus insecticide [135]. Notably, the same method has been used to detect Mycotoxins-Ochratoxin A in red wine and antibiotics (particularly chloramphenicol) in milk [3,23,136] to demonstrate its adaptability.

## 7. QDs-Based LFIA POC

**Figure 9:**
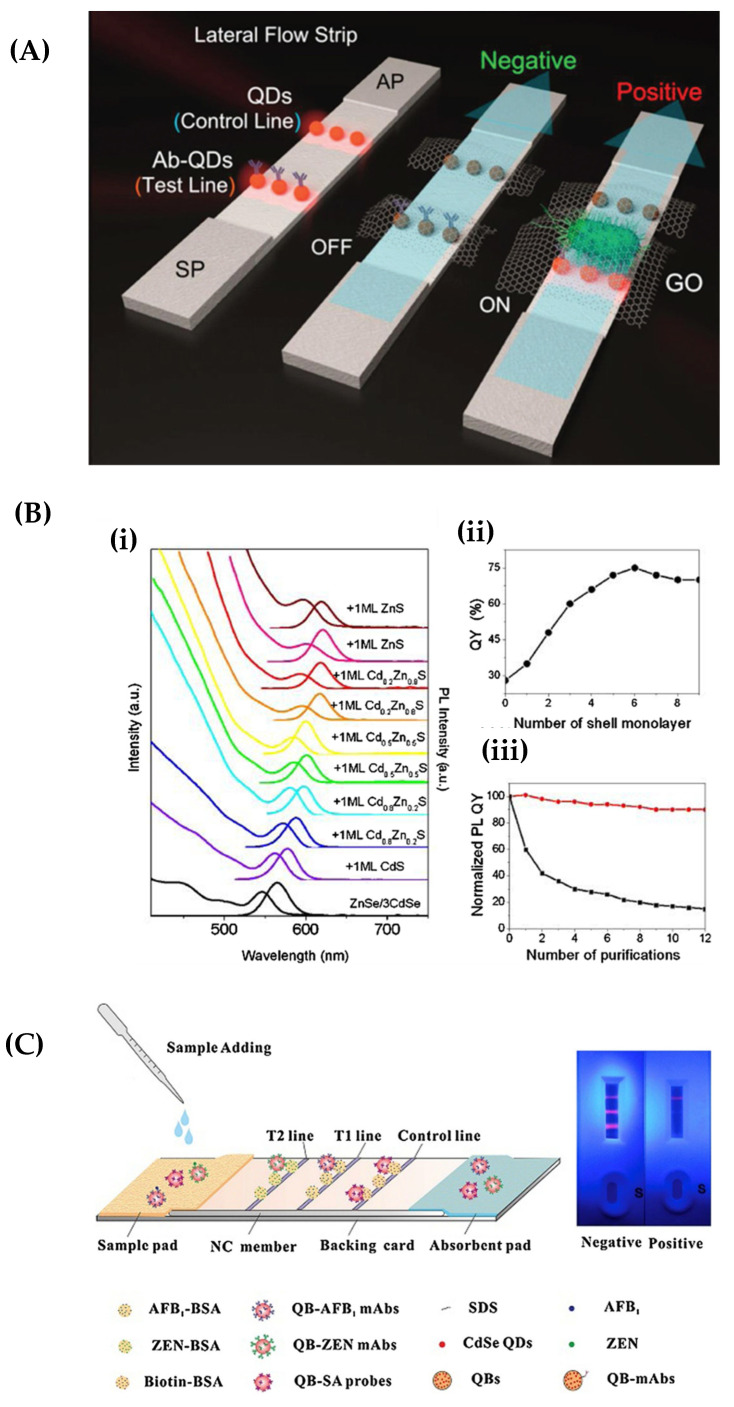
(**A**) Revelation of a quantum dot (QD)-based lateral flow immunoassay with graphene oxide (GO) for pathogen detection; absorbent pad (AP), antibody–quantum dot immunocomplex (Ab–QD), and sample pad (SP). Reproduced with permission from Ref. [157]. © 2019 American Dairy Science Association^®^. (**B**) (**i**) The absorption and photoluminescent spectra undergo progressive changes as the CdxZn1—xS composition is consecutively altered. (**ii**) The quantum yields exhibit a systematic evolution with each additional shell monolayer. (**iii**) The photoluminescent quantum yields display distinct variations following the successive precipitation of the ZnSe/3CdSe (represented by black squares) and ZnSe/3CdSe/CdxZn1—xS/ZnS (represented by red dots) core/shell QDs. Reproduced with permission from Ref. [158]. Copyright © 2023 Elsevier B.V. or its licensors or contributors. ScienceDirect^®^ is a registered trademark of Elsevier B.V (License Number: 5566100011830). (**C**) Procedure for the simultaneous detection of AFB_1_ and ZEN using QB-ICA. Reproduced with permission from Ref. [156]. Copyright © 2018 Elsevier B.V. All rights reserved (License Number: 5566081465711).

LFIA has emerged as a rapid point-of-care diagnostic tool, offering quick and efficient diagnoses. However, nanotechnology-based optimizations have been developed to enhance its performance and address its limitations [37,137,138]. A recent notable advancement in this field is the utilization of the QDs technique. QDs have attracted a lot of attention due to their extraordinary qualities, including exceptional quantum yields, extended fluorescence lifetimes, high extinction coefficients, and excellent stability [30,31]. QDs are a suitable choice for use as reporters due to their unique properties. They can be functionalized with conjugate antibodies, making it easier to create highly sensitive LFIAs. Figure 9A demonstrates a QD-based LFIA for pathogen detection, showcasing the effective integration of QDs in the LFIA framework to enhance sensitivity and enable accurate diagnostic results. In LFIAs, QDs are used as reporters alongside two specific antibodies. The capture antibody and conjugate antibody both target the analyte of interest. The conjugate antibodies, tagged with QDs, are immobilized on a conjugation pad, whereas the capture antibodies are fixed on a nitrocellulose membrane. The interaction between the QD-labeled antibody–target–antibody complex and the analyte produces a bright fluorescent band when exposed to UV light. This fluorescence can be used as a quantitative indicator for the presence of the analyte of interest.

Since 2010, there have been significant advancements in the development of quantum dot (QD)-based lateral flow immunoassays (LFIA) for point-of-care testing (POCT). These assays have shown great potential in detecting various entities such as viruses [139,140], proteins [29,141,142], nucleic acid [136], and medicine materials [33,143], with high sensitivity and specificity. LFIAs, using QD labels, have been found to outperform traditional colloidal gold labels, but with lower detection limits [139,144]. Shen et al. presented an innovative method for producing multishell nano-particles, which improved the sensitivity of the LFIA method [145]. Researchers were able to dramatically increase fluorescence quantum yields, and they successfully reduced exciton leakage by enclosing a ZnSe/3CdSe core in a CdS/CdxZn1-xS/ZnS multishell. In accordance with Figure 9B, the core/shell QD system increased fluorescence quantum yields and reduced exciton leakage, achieving fluorescence quantum yields ranging from 28% to 75%. This core/shell QD system exhibited exceptional stability across diverse physical conditions and a wide pH range. The modified QDs were able to detect the human hepatitis B surface antigen (HBsAg) with a sensitivity of 0.05 ng/mL. This innovative approach has led to the development of CdSe/ZnS QDs, which are now used as efficient labels in fluorescence-based lateral flow immunoassays [146,147,148]. In the study by Shen et al. [149], QD synthesis was further developed using photoluminescent CuInZnxS2 + x/ZnS materials, offering prospects for improved QD-based technologies. The core/shell QDs achieved a remarkable relative quantum yield of over 40% in water using an organic-aqueous phase transfer method, expanding their potential applications in various fields. These groundbreaking findings pave the way for further research and advancements in nanotechnology. Wu et al. [150] developed CIZS/ZnS/ZnS QDs using a two-step shell technique, achieving excellent quantum yields. These double-shell QDs allowed the quantitative detection of the C-reactive protein (CRP) to 5.8 ng/mL, showcasing their potential for highly specific and sensitive analytical procedures. Similarly, Huang et al. [151] introduced an innovative pitaya-type silica porous structure housing high-density QDs, enabling a robust fluorescence-based lateral flow immunoassay platform. This breakthrough allowed the accurate quantification of CRP concentrations in a broad range from 0.125 to 300 ng/mL, revolutionizing the field of diagnostic techniques. In their study, Anfossi et al. [152] introduced a groundbreaking technique, fluorescence-quenching LFIA (FQLFIA), for detecting fumonisin mycotoxins in maize flour. Using metal nanoparticles as quenchers, the method showed remarkable selectivity and sensitivity. When target analytes were present, the quencher displacement led to the recovery of fluorescence intensity in the QD labels. Additionally, a core/shell structure developed through cadmium-free synthesis offered an environmentally friendly and advanced alternative [150,153,154].

Foubert et al. [155] explored the difference between colloidal gold and quantum dots (QDs) for the simultaneous detection of mycotoxins. QD-based lateral flow immunoassays (LFIA) proved to be more sensitive and efficient. Moreover, Taranova et al. [130] introduced a groundbreaking test strip design featuring three color lines, akin to a traffic light, to offer a visually intuitive and easily interpretable format. In a groundbreaking study by Shao et al. [156], a cutting-edge technique utilizing QD nanobeads (QB) was introduced for a multiplexed immunochromatographic assay (QB-ICA). This innovative approach targeted the detection of crucial mycotoxins, ZEN and aflatoxin B1 (AFB1), which have significant implications for food safety and public health (as illustrated in Figure 9C). The researchers employed a streptavidin (SA)-biotin system for visualizing fluorescent signals, resulting in accurate and reliable results. The QB-ICA system exhibited exceptional precision when quantitatively analyzing AFB1 and ZEN levels by analyzing signal intensity ratios on two test lines (TLs) and the control line (CL). Remarkably, the system achieved impressively low detection limits of 1.65 pg/mL for AFB1 and 59.15 pg/mL for ZEN, highlighting its exceptional sensitivity and performance when identifying and quantifying these mycotoxins, even at minute concentrations.

## 8. QD-LFIA Testing for COVID-19

QD-LFIA is an innovative diagnostic test that has been developed for the detection of COVID-19. This testing method combines the principles of lateral flow immunoassay technology with the use of quantum dots, which are fluorescent nanoparticles, to provide rapid and accurate results. The QD-LFIA testing procedure begins with the collection of a sample, typically obtained through a nasal or throat swab. The collected sample is then mixed with a specific reagent that contains antibodies designed to target the SARS-CoV-2 virus, which causes COVID-19 [159,160]. These antibodies are conjugated with quantum dots, which are nanoscale particles capable of emitting fluorescent light signals when excited. Once the sample and reagent are combined, the mixture is applied to a test strip. The strip consists of several membranes that act as channels for the flow of the sample [161,162,163]. These membranes are pre-coated with different components, including a capture line and a control line. As the sample flows through the strip, it encounters the capture line, which contains immobilized viral antigens. If the SARS-CoV-2 virus is present in the sample, the quantum dot-conjugated antibodies bind to the viral antigens, forming a complex. This complex is then captured at the capture line, resulting in the accumulation of quantum dots at that location [164,165,166,167]. The control line, on the other hand, contains immobilized antibodies that can bind to the conjugated antibodies, regardless of the presence of the virus. It serves as a verification that the test is functioning correctly and that an adequate amount of sample has flowed through the strip. After a designated incubation period, typically ranging from 10 to 20 min, the test strip is examined for the presence of signals. The quantum dots, which have accumulated at the capture line, emit fluorescent light that can be detected and analyzed using a portable reader or a smartphone application. The intensity of the fluorescent signal is proportional to the number of viral antigens present in the sample, allowing for the quantitative measurement of the infection. QD-LFIA testing offers several advantages over traditional testing methods [159,168,169,170,171]. Firstly, it provides rapid results, usually within 15 to 30 min, allowing for quick decision-making and prompt patient management. Secondly, QD-LFIA has demonstrated high sensitivity and specificity when detecting the SARS-CoV-2 virus, ensuring the accurate identification of infected individuals [91,159,166,172]. Additionally, QD-LFIA testing is relatively simple to perform, making it suitable for use in various healthcare settings, including resource-limited environments and point-of-care settings. Its portability and low-cost nature contribute to its versatility and potential for widespread deployment. However, it is important to note that QD-LFIA testing is still undergoing further research and validation studies to ensure its accuracy and reliability when detecting COVID-19. As with any diagnostic test, there can be limitations, such as the potential for false-positive or false-negative results. Ongoing studies aim to optimize the test’s performance and address these challenges [166,168,173,174]. Wang and colleagues have developed a highly efficient colorimetric-fluorescent dual-mode lateral flow immunoassay (LFIA) biosensor, designed for the rapid and sensitive detection of SARS-CoV-2-specific IgM and IgG in human sera. This innovative biosensor employs spike (S) protein-conjugated SiO2@Au@QD nano-beads (NBs) as labels. To validate their method, the researchers utilized 16 positive serum samples from COVID-19 patients and 41 negative samples from patients with other viral respiratory infections. The results were remarkable, demonstrating that the SiO2@Au@QD LFIA combined detection method can successfully identify 100% of SARS-CoV-2 infected patients with 100% specificity [175].

## 9. Performance of the QD-Based LFIA as a POC Test for COVID-19 Detection

The performance of quantum dot-based lateral flow immunoassays (QD-based LFIA) has emerged as a promising point-of-care testing method for detecting COVID-19, offering several notable advantages in the battle against the pandemic. One of its key strengths lies in its high sensitivity, made possible by incorporating quantum dots into the assay. These quantum dots possess unique optical properties, such as intense and stable fluorescence, which significantly improve the signal-to-noise ratio, even allowing for the precise and accurate detection of low concentrations of viral antigens. This heightened sensitivity enables the early identification of COVID-19 infections, facilitating swift intervention, isolation, and treatment, thus curbing transmission rates. Moreover, the QD-based LFIA exhibits excellent specificity during COVID-19 detection. By utilizing specific antibodies that specifically bind to SARS-CoV-2 viral antigens, the test can differentiate between COVID-19 and other respiratory infections, minimizing the risk of false-positive or false-negative results. This specificity is crucial for accurate diagnoses, as it ensures that individuals infected with COVID-19 are correctly identified, and that appropriate measures are taken to prevent the further spread of the virus. Another vital characteristic of the QD-based LFIA is its rapid turnaround time, providing results within minutes. This quick response is particularly valuable in settings like healthcare facilities, airports, and community testing centers, as it enables immediate decision-making and reduces patient waiting times. Additionally, the user-friendly operation of the assay requires minimal technical expertise, making it suitable for deployment even in resource-limited or remote areas. The test involves straightforward steps, such as applying the sample to the test strip and observing the color change or fluorescent signal, eliminating the need for complex instrumentation or laboratory facilities. Although the performance of the QD-based LFIA is promising, it is essential to acknowledge the need for further validation and optimization. Large-scale clinical studies are necessary to evaluate the assay’s performance across diverse populations, accounting for varying viral loads, ages, and underlying health conditions. Additionally, research efforts must consider the impact of viral variants on the test’s sensitivity and specificity to ensure its ongoing effectiveness (Table 2) [159,167,168,170,176]. Wang et al. [177] responded to the WHO’s COVID-19 pandemic declaration by introducing a groundbreaking QD-LFIA method for the simultaneous detection of SARS-CoV-2 spike (S) and nucleocapsid (N) antigens. The method utilized a magnetic quantum dot, known as MagTQD, which featured a unique triple QD shell to amplify fluorescence signals under 365 nm UV light excitation. This ingenious coupling technique involved monoclonal antibodies targeting N and S antigens, which successfully conjugated onto MagTQD’s surface using EDC/NHS coupling chemistry. The QD-LFIA demonstrated remarkable sensitivity, with a detection limit of 1 pg/mL in direct mode, and 0.5 pg/mL in enrichment mode. Offering a high-performance diagnostic tool, this innovative approach enables the rapid and accurate detection of SARS-CoV-2 antigens, providing comprehensive test outcomes within 10 min in direct mode, and enhanced sensitivity within 20 min in enrichment mode. Moreover, the versatility of LFIA devices extends beyond COVID-19 diagnosis. As a prevalent sexually transmitted infection (STI), syphilis prompted the development of effective diagnostic methods to combat its global health implications. In a pivotal study conducted in 2010, Yang et al. [139] introduced a groundbreaking innovation known as the quantum dot-based lateral flow immunoassay (QD-LFIA) for syphilis screening. This pioneering assay utilized Cadmium Telluride (CdTe) quantum dots, which emitted unique light properties visible under a portable ultraviolet lamp. The objective was to detect anti-TP47 polyclonal antibodies, as their presence indicates syphilis infection. To achieve this, the researchers introduced thioglycolic acid (TGA) as a crucial linking agent, facilitating the interaction between quantum dots and Staphylococcal Protein A (SPA). The quantum dot-conjugated SPA formed a robust complex with the target antibodies, creating a bridge to the detection system. The next step involved recognizing and capturing the TP47 antigen that was immobilized on the test line, generating a distinct signal under ultraviolet light upon binding with the SPA-antibody complex. With a rapid 10 minute turnaround time, and remarkable sensitivity in detecting concentrations as low as 2 ng/mL, the QD-LFIA exhibited an exceptional performance. The evaluation, using 50 serum samples, showed 100% sensitivity and specificity, positioning the QD-LFIA as a highly promising alternative to the AuNPs-based method for syphilis detection.

## 10. Conclusions and Perspectives

In conclusion, the QD-based LFIA stands as a highly promising and innovative point-of-care testing method for the detection of COVID-19. By seamlessly integrating quantum dots into the traditional lateral flow assay format, this cutting-edge approach offers numerous advantages that significantly contribute to its effectiveness and potential impact with regard to combating the pandemic. First, the incorporation of quantum dots resulted in a remarkable improvement in terms of signal detection. Quantum dots possess exceptional optical properties, such as high brightness and photostability, leading to unparalleled sensitivity and enhanced detection limits compared with conventional lateral flow assays. This heightened sensitivity ensures the accurate identification of even minuscule viral loads, bolstering the precision and reliability of COVID-19 diagnoses. Furthermore, the quantum dot technology empowers the assay with multiplexing capabilities, making it possible to simultaneously detect multiple viral targets within a single test. This aspect is especially crucial given the context of COVID-19, wherein identifying different viral strains or co-infections can be paramount for devising effective treatment and containment strategies. By incorporating target-specific antibodies labeled with distinct quantum dots, the LFIA can provide comprehensive information concerning the presence and types of viral antigens, thereby empowering healthcare professionals to make more informed decisions. Apart from its sensitivity and multiplexing benefits, the QD-based LFIA excels in terms of convenience and speed, which are essential characteristics for point-of-care testing. The assay’s simple and user-friendly design allows for rapid and straightforward operation, making it highly suitable for deployment in various healthcare settings, including clinics, hospitals, and remote areas with limited access to sophisticated laboratory facilities. The ability to obtain quick results at the point of care is crucial for timely intervention, isolation, and treatment, thereby reducing the spread of the virus and substantially improving patient outcomes. Although the QD-based LFIA shows immense promise, it is essential to acknowledge the importance of further research and validation. Additional studies are necessary to optimize its performance in real-world conditions and validate its accuracy, specificity, and reliability. Rigorous evaluations will ensure its effectiveness across diverse populations and address potential challenges posed by new viral variants. Moreover, addressing scalability and cost considerations will be pivotal for enabling the large-scale production and widespread implementation of the assay. Ensuring affordability and accessibility will be key to its successful adoption in various regions, both in developed and developing countries. The QD-based LFIA represents a groundbreaking advancement in point-of-care COVID-19 testing. Its integration of quantum dot technology not only enhances sensitivity, but it also enables multiplexing capabilities while offering the advantages of simplicity and rapidity. With further development, validation, and optimization, this approach holds the potential to revolutionize COVID-19 detection, facilitating timely diagnosis and effective disease management, ultimately leading to improved global public health outcomes. Embracing such innovative and transformative technologies is crucial in our collective fight against infectious diseases, and the QD-based LFIA is poised to play a crucial role in this ongoing battle against COVID-19.

## Figures and Tables

**Table 1:** Applications of LFIA in the clinical field.

Application Field	Target	Matrix	Coefficient of Variation	Links
Health status biomarkers	Cardiac biomarker	Finger blood	8%–15%	[72]
		Serum	2.3%–8.4%	[73]
Viruses	SARS-CoV-2	Serum	-	[74]
		Serum	7.72%–9.66%	[75]
		Finger prick blood	<5%	[76]
		Saliva and serum	-	[77]
	Influenza A/B	Nasopharyngeal (nasal) swab	-	[78]
	Ebola	Blood	6.9%	[79]
Infectious diseases	Sepsis	Serum	5.92%–8.87%	[80]
	Candidiasis	Pharyngeal swabs	-	[81]

**Table 2:** Example of the performance of the QD-Based LFIA.

Type of QDs	Size of QDs	Pathogens	Targets	Performance	Ref.
CdSe/ZnS	15–20 nm	Mycobacterium tuberculosis	FprA antigens	LoD of 12.5 pg/μL in less than 10 min.	[178]
CdSe/ZnS QDs	Not reported	Fumonisin mycotoxins	----------	Visual LOD: 1.56–6.25 ng mL^−1^	[152]
Qdot	Not reported	Escherichia coli	Whole cells	LoD of 300 bacterial cells.	[144]
CdSe/ZnS	25 nm	Influenza A	Nucleoprotein antigens	100% accuracy and LoD of 0.016 HAU for H5 and 0.25 HAU for H9 in 15 min.	[179]
Cu:Zn−In−S/ZnS	Not reported	Clostridium tetani	Tetanus antibody	LoD of 0.001 IU/mL in 30 min.	[180]
CdTe QDs	Not reported	Vibrio parahaemolyticus	Culture media grown bacterial antigen	5.03 × 104 cfu L^−1^	[181]
CdTe	Not reported	Influenza A	Influenza A virus subtype H5 antigens	LoD of 0.09 ng/mL. Turnaround time in 10 min; 100% sensitivity and 88.2% specificity.	[140]

## Data Availability

All data generated or analyzed during this study are included in this published article.
